# Influencing Factors and Prediction Model Construction of Anxiety and Depression in Patients With Coronary Heart Disease Undergoing In-Stent Restenosis

**DOI:** 10.62641/aep.v54i4.2249

**Published:** 2026-08-15

**Authors:** Sihui Sun, Xiaoxian Wu, Lichen Tang, Haiyan Jiang

**Affiliations:** ^1^Department of Cardiology, Nanjing First Hospital, Nanjing Medical University, 210000 Nanjing, Jiangsu, China

**Keywords:** coronary disease, anxiety, depression, prediction model

## Abstract

**Background:**

In-stent Restenosis (ISR) represents a major challenge in interventional cardiology, and psychological distress is known to worsen coronary heart disease outcomes. However, the prevalence and determinants of anxiety and depression specifically in ISR patients remain largely uncharacterized. This study aimed to investigate the incidence and independent risk factors of these symptoms and to develop predictive models for their early identification.

**Methods:**

A single-center retrospective study involving 256 patients with confirmed ISR was conducted. Psychological status was assessed using the Self-Rating Anxiety Scale (SAS) and Self-Rating Depression Scale (SDS). Univariate and multivariate logistic regression analyses were employed to identify independent risk factors. Nomograms were constructed and validated based on these factors, with model performance evaluated by the area under the curve (AUC), calibration plots, and Decision Curve Analysis (DCA).

**Results:**

The prevalence of anxiety and depression symptoms was 37.89% and 42.58%, respectively. Multivariate analysis identified poorer sleep quality (higher Pittsburgh Sleep Quality Index (PSQI) score), lower social support (lower Social Support Rating Scale (SSRS) score), higher New York Heart Association (NYHA) classification (≥Ⅲ), multiple stents (≥2), and suburban/rural residence as independent risk factors for anxiety. For depression, older age, higher PSQI score, lower SSRS score, multiple stents, and advanced Mehran classification (III/IV) were significant predictors. The nomograms demonstrated good predictive accuracy, with AUCs of 0.79 (95% confidence interval (CI): 0.74–0.85) for anxiety and 0.82 (95% CI: 0.76–0.87) for depression, along with good calibration and clinical utility.

**Conclusions:**

Anxiety symptoms and depressive symptoms are highly prevalent in ISR patients. The developed nomograms, incorporating clinical and psychosocial factors, provide effective tools for individualized risk stratification, facilitating early identification and targeted psychological interventions to improve patient outcomes.

## Introduction

Coronary heart disease (CHD) remains the leading cause of cardiovascular mortality worldwide [1]. Its prevalence and incidence have been steadily increasing due to population aging and unfavorable lifestyle modifications, imposing a significant public health burden in China [2]. Percutaneous Coronary Intervention (PCI) has become the mainstay revascularisation strategy and markedly improves clinical outcomes for CHD patients [3]. However, In-stent Restenosis (ISR) continues to be an intractable clinical bottleneck in contemporary interventional cardiology [4]. Despite the significant reduction in ISR incidence achieved with drug-eluting stents, the rate remains at approximately 5–10%, constituting a patient cohort that cannot be overlooked [5, 6]. ISR not only elevates the risk of repeat revascularisation but also increases the likelihood of major adverse cardiac events, including myocardial infarction and cardiac death [7].

Mounting evidence supports a close link between cardiovascular disease and psychological factors [8, 9]. A review reported that the prevalence of anxiety and depression among CHD patients can range from 20% to 30%, with a substantially higher rate of 30% to 60% among survivors of acute myocardial infarction [10]. These psychological comorbidities independently worsen acute coronary syndrome prognosis and increase readmission and all-cause mortality [11]. For patients suffering from ISR, recurrent vascular lesions represent a severe psychological stressor, triggering persistent treatment uncertainty, health anxiety, and substantial economic and psychological burdens [12]. Anxiety and depression are closely linked to adverse cardiovascular outcomes, potentially exacerbating myocardial ischemia through behavioral and physiological pathways, forming a bidirectional cardiac-psychological vicious cycle [13]. Therefore, clarifying the occurrence patterns and risk factors of anxiety and depression in patients with ISR is of great clinical value for optimizing patient management.

Notably, most existing research focuses on general CHD or first-time PCI patients, while dedicated studies on ISR patients remain scarce. This high-risk subgroup lacks targeted data on psychological distress and its predictors. To address this research gap, this retrospective study investigated the risk factors of anxiety and depressive symptoms among ISR patients, integrating demographic, clinical, and psychosocial factors (including social support). This study aimed to establish evidence for personalised integrated care to improve the long-term quality of life and clinical prognoses for ISR patients.

## Materials and Methods

### Patient Population

This study was designed as a single-centre, retrospective, observational study. A total of 256 consecutive patients diagnosed with ISR of coronary arteries and readmitted for treatment at Nanjing First Hospital, Nanjing Medical University between June 2022 and May 2025 were enrolled. All patients were hospitalized due to recurrent angina symptoms or imaging studies indicating target vessel restenosis. This study was conducted in strict accordance with all the principles of the Declaration of Helsinki. This study was approved by the Medical Ethics Committee of Nanjing First Hospital, Nanjing Medical University (Approval Number: KY20260108-KS-01), and informed consent has been obtained from all enrolled patients.

### Inclusion and Exclusion Criteria

#### Inclusion Criteria

(1) aged ≥ 18 years; (2) diagnosed with ISR in accordance with established clinical diagnostic criteria [14]: coronary angiography confirms ≥50% luminal diameter stenosis within the stented segment or within 5 mm of the stent margins, accompanied by at least one of the following: (a) Recurrent angina symptoms; (b) Objective evidence of myocardial ischaemia (e.g., resting or stress electrocardiographic abnormalities); (c) Fractional flow reserve (FFR) < 0.8 (if measured); (d) Intravascular ultrasound (IVUS) demonstrating a minimum lumen cross-sectional area < 4 mm^2^ (if measured); (e) Asymptomatic stenosis with a lumen diameter reduction > 70%; (3) patients with complete and accessible electronic medical records.

#### Exclusion Criteria

(1) Combined with severe liver and kidney dysfunction; (2) Patients with malignant tumours or an estimated survival time of less than one year; (3) Patients who have participated in other clinical trials within the preceding three months; (4) Pregnant or lactating female patients; (5) Patients with a documented medical history of anxiety, depression, or other psychiatric disorders.

Initially, a total of 335 patients were screened for eligibility. Among them, 43 were excluded due to incomplete medical records or missing psychological assessment data; another 22 were excluded due to severe liver or kidney dysfunction, and 14 excluded due to malignant tumours. Ultimately, 256 patients with angiographically confirmed ISR were enrolled for the final analysis.

### Data Collection

All clinical and psychological data were retrospectively collected and extracted from the hospital electronic medical record system by two independently trained researchers using a standardized data extraction form. All data had been routinely generated and documented during clinical practice prior to study initiation, and no new assessments or interventions were performed for the purpose of this study. The collected variables included: (1) demographic characteristics: age, gender, educational level, marital status, and residence. (2) clinical data: history of hypertension, diabetes, hyperlipidaemia, New York Heart Association (NYHA) functional classification (a standard grading system for assessing heart failure severity, class I–IV) [15], left ventricular ejection fraction (LVEF), number of stents implanted, and Mehran classification (a 4-grade angiographic scoring system evaluating ISR lesion severity and complexity: I–IV) of ISR [16]. (3) psychosocial and behavioural measures: scores from the Self-Rating Anxiety Scale (SAS), Self-Rating Depression Scale (SDS), Pittsburgh Sleep Quality Index (PSQI), and Social Support Rating Scale (SSRS). Any discrepancies in data extraction were resolved through discussion with a third senior cardiologist to guarantee the accuracy and reliability.

### Psychological and Behavioural Assessment

All participants underwent standardized psychological and behavioural assessments using validated Chinese versions of the following scales:

(1) SAS [17]: The SAS is a 20-item self-administered questionnaire designed to assess the severity of anxiety symptoms over the preceding week. Each item is scored on a 4-point Likert scale, ranging from 1 (“a little of the time”) to 4 (“most of the time”). The total score, ranging from 20 to 80, is converted to a standard score by multiplying the raw sum by 1.25. Higher standard scores indicate more severe anxiety symptoms. A standard score ≥50 is considered clinically significant for anxiety, with severity categorized as mild (50–59), moderate (60–69), and severe (≥70) levels [18]. This cutoff is widely used and widely applied in Chinese clinical populations for screening clinical anxiety. The Chinese version of the SAS has exhibited good reliability and validity in various patient populations [19]. In the present study, the Cronbach’s α for the SAS was 0.84.

(2) SDS [20]: The SDS comprises 20 items that assess affective, psychological, and somatic symptoms of depression over the past week. Items are rated on a 4-point scoring scheme as the SAS. The raw total raw score (20–80) is converted to a standard score by multiplying by 1.25. A standard score ≥53 suggests the presence of clinically relevant depressive symptoms, categorized as mild (53–62), moderate (63–72), and severe (≥73) [18]. This threshold is well-established and commonly adopted in Chinese cardiovascular patient populations. The Chinese version of the SDS has been widely used and validated [19]. The Cronbach’s α coefficient of the SDS in this cohort was 0.86, indicating excellent internal consistency.

(3) PSQI [21]: The PSQI is a 19-item self- administered questionnaire that assesses sleep quality and disturbances over a 1-month interval. It comprises seven subscales: subjective sleep quality, sleep latency, sleep duration, habitual sleep efficiency, sleep disturbances, use of sleeping medication, and daytime dysfunction. The sum of these component scores yields a global PSQI score ranging from 0 to 21, with higher scores indicating poorer sleep quality. A global score > 5 is defined as clinically significant sleep disturbance. The application of PSQI among the Chinese population has been widely verified [22]. The validated Chinese version was employed in this study, which demonstrated good internal consistency with a Cronbach’s α of 0.78.

(4) SSRS [23]: The SSRS is a 10-item scale designed to measure an individual’s perceived social support across three dimensions: subjective support (4 items), objective support (3 items), and support utilization (3 items). The total score ranges from 12 to 66, and higher scores reflecting stronger perceived social support. This scale is extensively -validated and commonly used in Chinese clinical and social research. For the present cohort, the SSRS exhibited a Cronbach’s α of 0.81, suggesting good internal consistency.

All scales have been widely used in Chinese clinical populations and have demonstrated good reliability and validity.

### Statistical Analysis

Statistical analyses were performed using SPSS version 26.0 (IBM Corp., Armonk, NY, USA). The Shapiro–Wilk test was used to assess the normality of continuous variables. Normally distributed continuous data were presented as mean ± standard deviation (SD) and compared using the independent samples t-test. Non normally distributed quantitative data were reported as median (interquartile range (IQR)) and analysed using the Mann–Whitney U test. Categorical variables were summarized as counts and percentages and compared using the chi-square test or Fisher’s exact test. Univariate analyses were first carried out to screen potential factors associated with anxiety and depression symptoms. Multicollinearity diagnostics were performed using the variance inflation factor (VIF) prior to multivariate regression analysis. A VIF < 5 was defined as the absence of significant collinearity. All variables with a *p* < 0.05 in univariate analysis were initially entered into the multivariate logistic regression model, and the forward stepwise selection procedure was then applied to identify independent risk factors based on the pre-specified entry and retention criteria of the model. Variables were selected based on clinical relevance and univariate significance to ensure model parsimony and interpretability. Results were expressed as odds ratios (OR) with 95% confidence intervals (CI). Based on the independent risk factors identified from the multivariate logistic regression, two nomograms were constructed to predict the probability of anxiety and depression, respectively. The discriminative ability of each nomogram was evaluated using the area under the curve (AUC). Calibration was assessed using the Hosmer–Lemeshow goodness-of-fit test and calibration plots. Decision Curve Analysis (DCA) was performed to evaluate the clinical utility of the models by quantifying the net benefit across a range of threshold probabilities. A two-tailed *p* < 0.05 was considered statistically significant.

## Results

### Baseline Prevalence of Anxiety and Depressive Symptoms

A total of 256 patients with ISR were included in this final analysis. As summarized in Table 1, the prevalence of anxiety symptoms (SAS score ≥50) was 37.89% (97/256), with the majority of cases being mild (87 cases). The incidence of depressive symptoms (SDS score ≥53) was slightly higher, at 42.58% (109/256), with 83, 25, and 1 case classified as mild, moderate, and severe, respectively.

**Table 1.** **Current status of anxiety and depression levels**.

Variables	Total	Mild	Moderate	Severe	Incidence rate^c^
SAS score^a^	256	87	10	0	37.89%
SDS score^b^	256	83	25	1	42.58%

^a^Mild: 50–59; Moderate: 60–69; Severe: ≥70. ^b^Mild: 53–62; Moderate: 63–72; Severe: ≥73. ^c^Incidence rate = (Mild + Moderate + Severe)/Total cases. SAS, Self-Rating Anxiety Scale; SDS, Self-Rating Depression Scale.

### Factors Associated With Anxiety Symptoms in Univariate Analysis 

Univariate analysis comparing patients with and without anxiety is presented in Table 2. There is no significant differences were observed between the two groups in terms of age, gender, education level, hypertension, diabetes, hyperlipemia, LVEF, or Mehran classification (*p* > 0.05). However, the anxiety group demonstrated significantly higher PSQI scores (worse sleep quality) and lower SSRS (social support) scores (*p* < 0.001). Furthermore, several clinical and social factors were significantly associated with anxiety, including marital status (higher risk in unmarried/divorced/widowed, *p* = 0.006), residence (higher risk in suburban/rural areas, *p* = 0.009), NYHA functional classification (higher risk in class ≥Ⅲ, *p* = 0.022), and implantation of stents quantity (higher risk with ≥2 stents, *p* = 0.002).

**Table 2.** **Univariate analysis of anxiety**.

Variables		Total (256 cases)	Non-anxiety (159 cases)	Anxiety (97 cases)	Statistic	*p*
Age (years), Mean ± SD		57.50 ± 7.02	57.06 ± 7.27	58.24 ± 6.55	t = –1.31	0.192
PSQI score (points), Mean ± SD		12.55 ± 3.54	11.75 ± 3.48	13.87 ± 3.25	t = –4.83	<0.001
SSRS score (points), Mean ± SD		34.45 ± 4.25	35.32 ± 3.94	33.03 ± 4.38	t = 4.32	<0.001
Sex, n (%)					χ^2^ = 0.12	0.730
	Female	167 (65.23)	105 (66.04)	62 (63.92)		
	Male	89 (34.77)	54 (33.96)	35 (36.08)		
Degree of education, n (%)					χ^2^ = 0.41	0.521
	High school and above	149 (58.20)	95 (59.75)	54 (55.67)		
	Junior high school and below	107 (41.80)	64 (40.25)	43 (44.33)		
Marital status, n (%)					χ^2^ = 7.49	0.006
	Married	200 (78.12)	133 (83.65)	67 (69.07)		
	Unmarried/Divorced/widowed	56 (21.88)	26 (16.35)	30 (30.93)		
Residence, n (%)					χ^2^ = 6.91	0.009
	Urban	148 (57.81)	102 (64.15)	46 (47.42)		
	Suburban/Rural	108 (42.19)	57 (35.85)	51 (52.58)		
Hypertension, n (%)					χ^2^ = 1.66	0.197
	Negative	98 (38.28)	56 (35.22)	42 (43.30)		
	Positive	158 (61.72)	103 (64.78)	55 (56.70)		
Diabetes, n (%)					χ^2^ = 0.36	0.550
	Negative	208 (81.25)	131 (82.39)	77 (79.38)		
	Positive	48 (18.75)	28 (17.61)	20 (20.62)		
Hyperlipemia, n (%)					χ^2^ = 3.70	0.054
	Negative	159 (62.11)	106 (66.67)	53 (54.64)		
	Positive	97 (37.89)	53 (33.33)	44 (45.36)		
NYHA classification, n (%)					χ^2^ = 5.27	0.022
	<Ⅲ	160 (62.50)	108 (67.92)	52 (53.61)		
	≥Ⅲ	96 (37.50)	51 (32.08)	45 (46.39)		
LVEF, n (%)					χ^2^ = 0.65	0.422
	≥50%	222 (86.72)	140 (88.05)	82 (84.54)		
	<50%	34 (13.28)	19 (11.95)	15 (15.46)		
Number of stents, n (%)					χ^2^ = 9.75	0.002
	1	196 (76.56)	132 (83.02)	64 (65.98)		
	≥2	60 (23.44)	27 (16.98)	33 (34.02)		
Mehran classification, n (%)					χ^2^ = 3.14	0.076
	Ⅰ and Ⅱ	129 (50.39)	87 (54.72)	42 (43.30)		
	Ⅲ and Ⅳ	127 (49.61)	72 (45.28)	55 (56.70)		

PSQI, Pittsburgh Sleep Quality Index; SSRS, Social Support Rating Scale; NYHA, New York Heart Association; LVEF, left ventricular ejection fraction; SD, standard deviation.

### Independent Risk Factors and Prediction Model for Anxiety Symptoms

No significant multicollinearity was observed among candidate variables (all VIF < 5). Variables with significance in the univariate analysis were subsequently included in a multivariate logistic regression model to identify independent predictors of anxiety. As shown in Table 3, higher PSQI score (OR = 1.24, 95% CI: 1.13–1.37, *p* < 0.001), unmarried/divorced/widowed status (OR = 2.16, 95% CI: 1.09–4.28, *p* = 0.028), suburban/rural residence (OR = 2 .57, 95% CI: 1.40–4.71, *p* = 0.002), NYHA classification ≥Ⅲ (OR = 2.19, 95% CI: 1.19–4.02, *p* = 0.011), and implantation of stents quantity ≥2(OR = 2.72, 95% CI: 1.38–5.37, *p* = 0.004) were independent risk factors for anxiety. Conversely, a higher SSRS score was a protective factor (OR = 0.86, 95% CI: 0.80–0.93, *p* < 0.001).

**Table 3.** **Analysis of independent risk factors for anxiety**.

Variables		β	SE	Wald χ^2^	*p*	OR (95%CI)
PSQI score		0.22	0.05	19.52	<0.001	1.24 (1.13–1.37)
SSRS score		–0.15	0.04	15.45	<0.001	0.86 (0.80–0.93)
Marital status						
	Married					1.00 (Reference)
	Unmarried/Divorced/Widowed	0.77	0.35	4.86	0.028	2.16 (1.09–4.28)
Residence						
	Urban					1.00 (Reference)
	Suburban/Rural	0.94	0.31	9.36	0.002	2.57 (1.40–4.71)
NYHA classification						
	<Ⅲ					1.00 (Reference)
	≥Ⅲ	0.78	0.31	6.42	0.011	2.19 (1.19–4.02)
Number of stents						
	1					1.00 (Reference)
	≥2	1.00	0.35	8.38	0.004	2.72 (1.38–5.37)

PSQI, Pittsburgh Sleep Quality Index; SSRS, Social Support Rating Scale; NYHA, New York Heart Association; OR, odds ratio; CI, confidence interval; SE, standard error.

### Factors Associated With Depressive Symptoms in Univariate Analysis

The univariate analysis for factors associated with depression is detailed in Table 4. The depression group was significantly older and had higher PSQI scores and lower SSRS scores compared to the non-depression group (*p* < 0.001). Additionally, marital status (unmarried/divorced/widowed, *p* = 0.029), diabetes (*p* = 0.014), LVEF <50% (*p* = 0.040), number of stents ≥2 (*p* < 0.001), and Mehran classification (III and IV, *p* = 0.003) were significantly more prevalent in the depression group.

**Table 4.** **Univariate analysis of depression**.

Variables		Total (256 cases)	Non-depression (147 cases)	Depression (109 cases)	Statistic	*p*
Age (years), Mean ± SD		57.50 ± 7.02	56.26 ± 7.54	59.18 ± 5.87	t = –3.49	<0.001
PSQI score (points), Mean ± SD		12.55 ± 3.54	11.86 ± 3.50	13.49 ± 3.40	t = –3.71	<0.001
SSRS score (points), Mean ± SD		34.45 ± 4.25	35.85 ± 3.57	32.57 ± 4.38	t = 6.40	<0.001
Sex, n (%)					χ^2^ = 0.06	0.812
	Female	167 (65.23)	95 (64.63)	72 (66.06)		
	Male	89 (34.77)	52 (35.37)	37 (33.94)		
Degree of education, n (%)					χ^2^ = 0.14	0.712
	High school and above	149 (58.20)	87 (59.18)	62 (56.88)		
	Junior high school and below	107 (41.80)	60 (40.82)	47 (43.12)		
Marital status, n (%)					χ^2^ = 4.79	0.029
	Married	200 (78.12)	122 (82.99)	78 (71.56)		
	Unmarried/Divorced/widowed	56 (21.88)	25 (17.01)	31 (28.44)		
Residence, n (%)					χ^2^ = 0.60	0.440
	Urban	148 (57.81)	88 (59.86)	60 (55.05)		
	Suburban/Rural	108 (42.19)	59 (40.14)	49 (44.95)		
Hypertension, n (%)					χ^2^ = 2.66	0.103
	Negative	98 (38.28)	50 (34.01)	48 (44.04)		
	Positive	158 (61.72)	97 (65.99)	61 (55.96)		
Diabetes, n (%)					χ^2^ = 6.00	0.014
	Negative	208 (81.25)	127 (86.39)	81 (74.31)		
	Positive	48 (18.75)	20 (13.61)	28 (25.69)		
Hyperlipemia, n (%)					χ^2^ = 0.49	0.482
	Negative	159 (62.11)	94 (63.95)	65 (59.63)		
	Positive	97 (37.89)	53 (36.05)	44 (40.37)		
NYHA classification, n (%)					χ^2^ = 1.79	0.181
	<Ⅲ	160 (62.50)	97 (65.99)	63 (57.80)		
	≥Ⅲ	96 (37.50)	50 (34.01)	46 (42.20)		
LVEF, n(%)					χ^2^ = 4.23	0.040
	≥50%	222 (86.72)	133 (90.48)	89 (81.65)		
	<50%	34 (13.28)	14 (9.52)	20 (18.35)		
Number of stents, n(%)					χ^2^ = 11.68	<0.001
	1	196 (76.56)	124 (84.35)	72 (66.06)		
	≥2	60 (23.44)	23 (15.65)	37 (33.94)		
Mehran classification, n(%)					χ^2^ = 9.09	0.003
	Ⅰ and Ⅱ	129 (50.39)	86 (58.50)	43 (39.45)		
	Ⅲ and Ⅳ	127 (49.61)	61 (41.50)	66 (60.55)		

PSQI, Pittsburgh Sleep Quality Index; SSRS, Social Support Rating Scale; NYHA, New York Heart Association; LVEF, left ventricular ejection fraction.

### Independent Risk Factors and Prediction Model for Depressive Symptoms

Collinearity was not significant among included variables (all VIF < 5). Multivariate logistic regression analysis for depression (Table 5) identified older age (OR = 1.09, 95% CI: 1.04–1.14, *p* < 0.001), higher PSQI score (OR = 1.18, 95% CI: 1.08–1.30, *p* < 0.001), number of stents ≥2 (OR = 2.88, 95% CI: 1.41–5.84, *p* = 0.004), and Mehran classification (III and IV) (OR = 2.58, 95% CI: 1.40–4.72, *p* = 0.002) as independent risk factors. A higher SSRS score was again a significant protective factor (OR = 0.81, 95% CI: 0.75–0.88, *p* < 0.001) (Table 5).

**Table 5.** **Analysis of independent risk factors for depression**.

Variables		β	SE	Wald χ^2^	*p*	OR (95%CI)
Age		0.09	0.02	13.00	<0.001	1.09 (1.04–1.14)
PSQI score		0.17	0.05	12.53	<0.001	1.18 (1.08–1.30)
SSRS score		–0.21	0.04	27.66	<0.001	0.81 (0.75–0.88)
Diabetes						
	Negative					1.00 (Reference)
	Positive	0.58	0.38	2.30	0.129	1.79 (0.84–3.77)
LVEF						
	≥50%					1.00 (Reference)
	<50%	0.84	0.44	3.69	0.055	2.33 (0.98–5.51)
Number of stents						
	1					1.00 (Reference)
	≥2	1.06	0.36	8.52	0.004	2.88 (1.41–5.84)
Mehran classification						
	Ⅰ and Ⅱ					1.00 (Reference)
	Ⅲ and Ⅳ	0.95	0.31	9.33	0.002	2.58 (1.40–4.72)

PSQI, Pittsburgh Sleep Quality Index; SSRS, Social Support Rating Scale; LVEF, left ventricular ejection fraction; OR, odds ratio; CI, confidence interval; SE, standard error.

### Performance and Validation of the Anxiety Symptoms Prediction Model

**Fig. 1.**
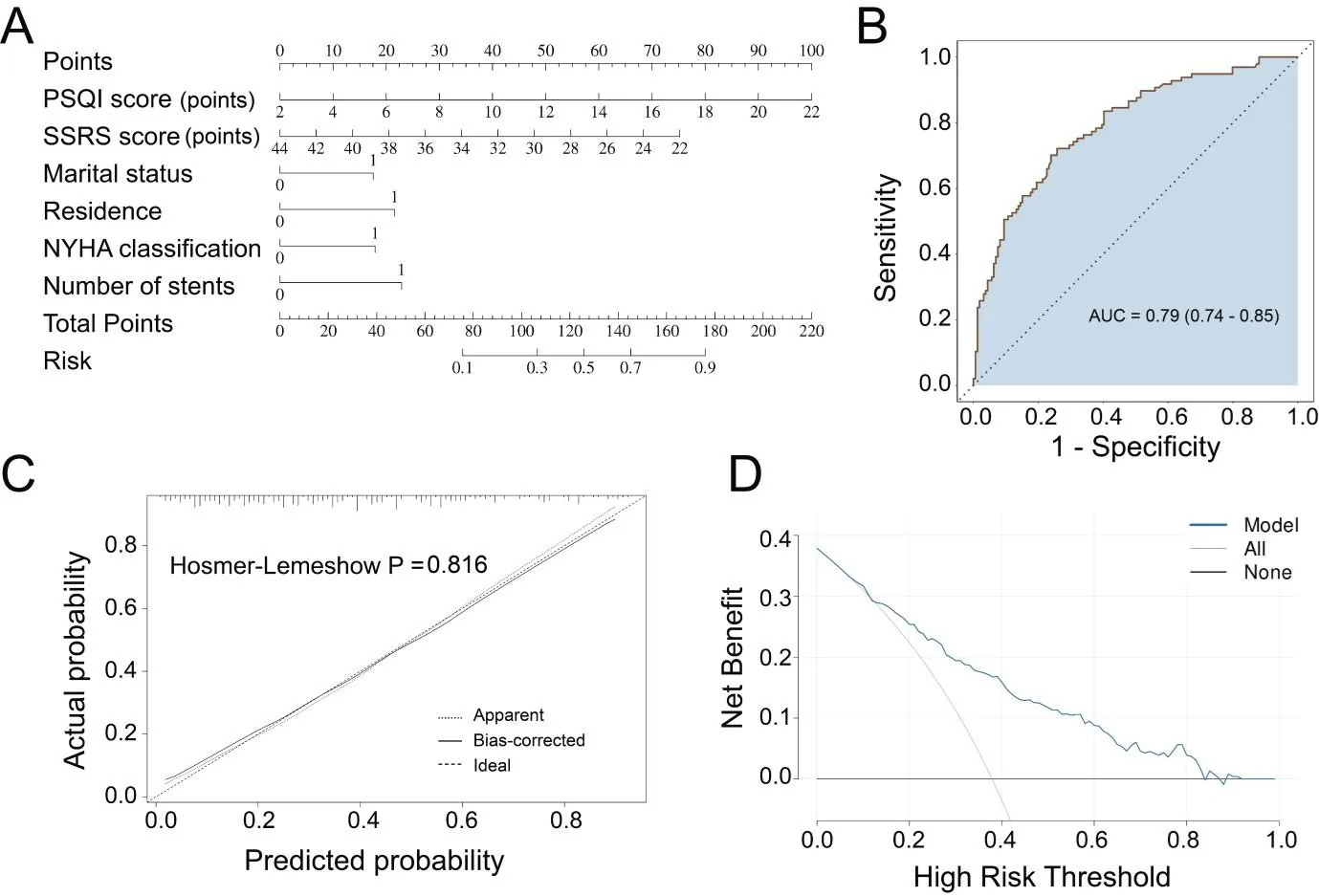
**Development and validation of the anxiety prediction nomogram**. (A) Nomogram (marital status: 1 = unmarried/divorced/widowed; residence: 1 = suburban/rural; NYHA classification: 1 = ≥Ⅲ; number of stents: 1 = ≥2 stents.). (B) ROC curve showing discriminative ability. (C) Calibration plot. (D) Decision curve analysis demonstrating clinical utility. PSQI, Pittsburgh Sleep Quality Index; SSRS, Social Support Rating Scale; NYHA, New York Heart Association; ROC, receiver operating characteristic; AUC, area under the curve.

A nomogram for predicting the probability of anxiety was constructed based on the six independent factors (Fig. 1A). The ROC curve demonstrated an AUC of 0.79 (95% CI: 0.74–0.85), indicating good discriminative ability (Fig. 1B). The calibration curve showed excellent agreement between the predicted probabilities and actual outcomes (Hosmer-Lemeshow test, *p* = 0.816) (Fig. 1C). DCA confirmed the clinical utility of the model, as it provided a positive net benefit across the threshold probability range of 0.11–0.83 (Fig. 1D), indicating that the nomogram is clinically useful for risk stratification and decision-making in most clinically relevant risk scenarios.

### Performance and Validation of the Depressive Symptoms Prediction Model

**Fig. 2.**
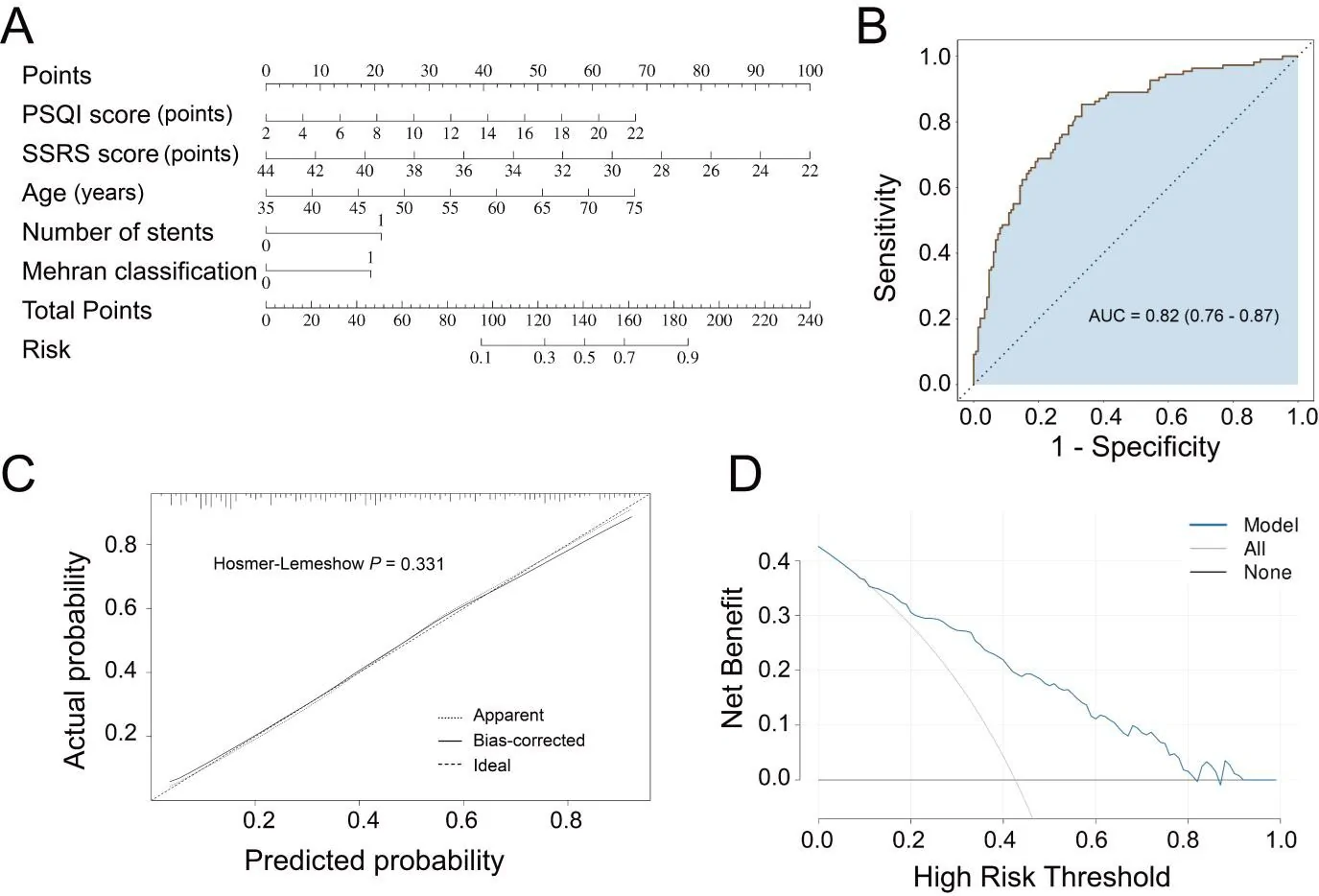
**Development and validation of the depression prediction nomogram**. (A) Nomogram (number of stents: 1 = ≥2 stents; Mehran classification: 1 = grade III/IV). (B) ROC curve showing discriminative ability. (C) Calibration plot. (D) Decision curve analysis demonstrating clinical utility. PSQI, Pittsburgh Sleep Quality Index; SSRS, Social Support Rating Scale; NYHA, New York Heart Association; ROC, receiver operating characteristic; AUC, area under the curve.

A nomogram incorporating age, PSQI score, SSRS score, number of stents, and Mehran classification was developed to visualize the risk of depression (Fig. 2A). The ROC analysis yielded an AUC of 0.82 (95% CI: 0.76–0.87), signifying good discrimination between patients with and without depression (Fig. 2B). The calibration plot also indicated a good fit (Hosmer-Lemeshow test, *p* = 0.331) (Fig. 2C). The DCA further validated the model’s clinical value, showing a positive net benefit within the threshold probability range of 0.12–0.81 (Fig. 2D), supporting its practical clinical application for individualized risk assessment.

## Discussion

This study systematically explored the prevalence and independent risk factors for anxiety and depression symptoms among patients with ISR. The development and validation of two individualised prediction models were successful, with the models demonstrating adequate performance. The main findings are summarized as follows: Firstly, anxiety and depressive symptoms are highly prevalent among ISR patients, with both conditions affecting over 35% of the cohort. Secondly, poorer sleep quality, lower social support, more severe clinical status (e.g., higher NYHA class, greater number of stents, poorer Mehran classification) and specific sociodemographic characteristics (e.g., unmarried/divorced/widowed status, rural residence) were identified as independent associated factors for anxiety and/or depression. The nomogram model constructed on the basis of these factors has demonstrated excellent discrimination, calibration, and clinical utility in predicting individual patients’ risk of anxiety and depression.

The incidence rates of anxiety and depression among ISR patients were reached 37.89% and 42.58% respectively, which was significantly higher than that in the general population [24] and among the highest rates reported for general CHD cohorts [25, 26]. This finding strongly suggests that ISR represents a major psychological stressor for patients. Notably, differences in study populations (general CHD versus ISR patients) and assessment tools may limit direct comparability across studies. Patients not only face recurrent angina symptoms but may also harbour doubts about the long-term efficacy of their initial revascularisation procedure [12]. They experience profound uncertainty and fear regarding their future health trajectory, alongside the financial strain from repeated interventional procedures [27]. This cumulative “second hit” effect makes ISR patients a special subgroup with extremely fragile psychology and in urgent need for targeted clinical intervention and regular psychological screening in this high-risk group.

A key and consistent finding of this study is that sleep quality (PSQI score) and social support (SSRS score) are common and powerful independent predictors of anxiety and depression among patients with ISR, as confirmed by both univariate and multivariate logistic regression analyses. This result aligns well with the bidirectional interaction theory of the “mind-body” axis [28, 29]. On one hand, anxiety and depressive symptoms may be closely associated with the onset and deterioration of insomnia and poor sleep quality. This relationship may be mediated by activation of the hypothalamic-pituitary-adrenal (HPA) axis and sympathetic nervous system, which further exacerbate inflammatory responses and endothelial dysfunction [30, 31]. On the other hand, sleep disturbances can further disrupt the autonomic nerve balance and hormone secretion, exacerbating daytime functional disorders and negative emotions, thus creating a vicious cycle [32]. Social support is regarded as a key protective resource, which can buffer disease stress, improve treatment compliance, and encourage positive health behaviors [33]. Consistent with this, a recent cross-sectional study by Won [34] also highlighted the protective role of social support in patients with coronary artery disease (CAD). Therefore, incorporating assessments of sleep quality and social support into the routine management of ISR patients and using them as potential targets for psychological intervention has significant clinical significance. Notably, sleep quality and social support are modifiable factors, which makes them highly actionable intervention targets for alleviating anxiety and depression in daily clinical practice.

In terms of clinical factors, we found that the implantation of stents quantity (≥2) was a shared independent risk factor for both anxiety and depression. This directly reflects the severity of the patient’s disease burden. More stents often imply more diffuse and severe coronary artery lesions, as well as a more complex clinical background, thereby bringing patients a stronger illness perception and concerns about the future. In a retrospective study by Tang *et al*. [35], an increase in the implantation of stents quantity was found to be an independent risk factor for ISR, which directly verified this inference. Higher NYHA classification (≥Ⅲ) reflects more severe cardiac dysfunction and greater limitation in physical activity. A study has suggested that patients with advanced functional impairment are more vulnerable to psychological distress, as persistent physical symptoms and reduced exercise capacity serve as constant reminders of disease severity [36]. Complementary to this, higher Mehran classification (Grade III–IV, indicating more severe and complex ISR lesions) is an independent predictor of depression. Mehran classification represents the complexity of ISR lesions under angiography [37]. More complex lesions are usually associated with difficult revascularization, a higher risk of recurrence, and a unfavourable prognosis. These uncertainties are more likely to trigger core depressive symptoms such as helplessness and hopelessness. Notably, Mehran grading correlated with depression rather than anxiety, since it reflects long-term prognostic uncertainty and disease chronicity, which are more closely linked to depressive mood. In contrast, NYHA classification was associated with anxiety but not depression, as it captures acute symptomatic limitation, physical discomfort, and immediate functional impairment, which are potent triggers for anxious distress and rumination.

In terms of sociodemographic characteristics, unmarried, divorced, widowed status and rural residency were identified as independent risk factors for anxiety. The absence of emotional support and practical care from a spouse undoubtedly weakens a patient’s ability to cope with the stress of the disease. Rural residents may encounter problems such as poor accessibility of medical resources, relatively lack of health literacy and heavier economic burdens, all of which can exacerbate psychological distress. It is worth noting that advanced age was an independent risk factor for depression, which may be related to the fact that elderly patients often have multiple chronic diseases, loss of social roles, increased loneliness, and cognitive decline. A key contribution of this study is the development and validation of anxiety and depression prediction nomograms for ISR patients. Both models exhibited good discrimination and favourable calibration, and DCA confirmed clinical net benefit. This provides a preliminary decision support tools for early psychological screening and targeted intervention. Nevertheless, external validation is required prior to wide-scale clinical implementation. Given the single centre and retrospective design, prospective and multicentre external validation in an independent cohort is essential before these nomograms can be applied in routine clinical practice. For instance, patients predicted as high-risk by the model could be referred early to psychiatric services or cardiac psychosocial rehabilitation programmes, receive enhanced patient education, and undergo proactive management of sleep disturbances.

Several limitations of study should be acknowledged. First, this is a single-centre retrospective study, and it cannot establish a causal relationship; therefore, it remains unclear whether the observed anxiety and depressive symptoms preceded the ISR onset or emerged as acute emotional reactions to it. Multicentre prospective studies with longitudinal follow-up are needed to address these issues. Second, all psychological outcomes were assessed via self-reported questionnaires. Although validated, they are susceptible to reporting bias. Future research may incorporate clinical interviews to enhance diagnostic accuracy. Additionally, multiple comparisons in univariate analyses were performed without correction for multiple testing, which may increase the risk of type I error—a common limitation in exploratory observational studies and requires cautious interpretation. Third, the cohort contained no cases of severe anxiety and only one case of severe depression, limiting generalisability to patients with severe psychological distress. Moreover, the prediction models were validated only internally; external validation in independent cohorts is essential before clinical application. Finally, the multivariate models adopted forward stepwise variable selection, which carries risks of overfitting and may exclude variables with small but clinically meaningful effects. Alternative strategies (e.g., Least Absolute Shrinkage and Selection Operator or theory-driven preselection) should be considered in future studies.

## Conclusions

This study identifies a high prevalence of anxiety and depressive symptoms among patients with ISR, underscoring a significant psychological burden. We have identified poor sleep quality, insufficient social support, severe clinical disease burden, and specific sociodemographic characteristics as key independent contributors to anxiety symptoms and depressive symptoms in this population through univariate and multivariate logistic regression analyses. The constructed and rigorously validated nomograms effectively integrate these readily accessible clinical available variables, providing clinicians with practical, individualized tools for early risk stratification. These models demonstrated good discrimination, calibration, and clinical net benefit. Implementing such predictive tools in clinical practice could facilitate the timely identification of well-validated, enabling targeted interventions such as psychological counseling, sleep optimization, and improved social support management. Ultimately, a comprehensive management strategy that addresses both psychological and physical health is crucial for improving the long-term prognosis and quality of life in patients facing ISR.

## Availability of Data and Materials

All data included in this study can be obtained by contacting the corresponding author if needed.
